# A Bright Future? A Perspective on Class C GPCR Based Genetically Encoded Biosensors

**DOI:** 10.1021/acschemneuro.3c00854

**Published:** 2024-02-21

**Authors:** Margulan Otanuly, Martin Kubitschke, Olivia Andrea Masseck

**Affiliations:** Synthetische Biologie, Universität Bremen, Bremen 28359, Germany

**Keywords:** Molecular neuroscience, neuromodulator dynamics, class C GPCR, biosensors, GPCR-based biosensors, neurotransmitters

## Abstract

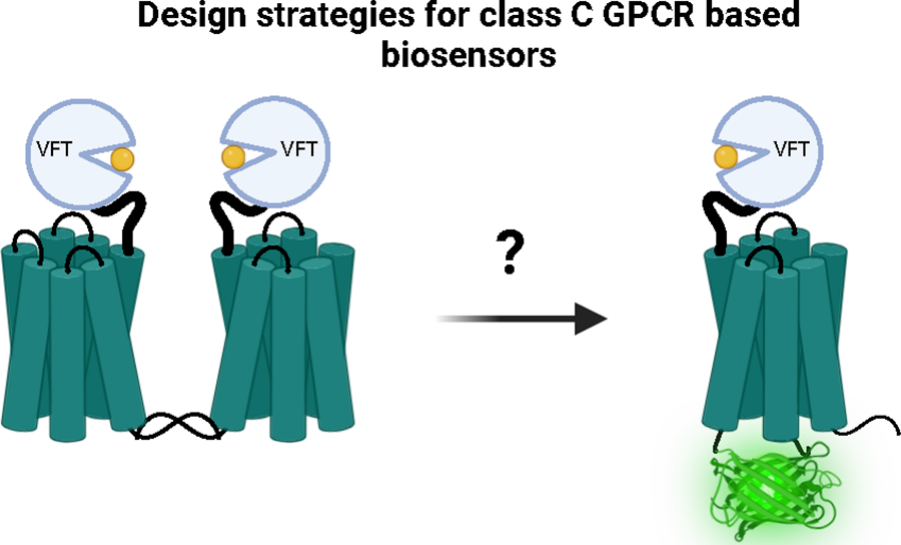

One of the major challenges in molecular neuroscience today is to accurately monitor neurotransmitters, neuromodulators, peptides, and various other biomolecules in the brain with high temporal and spatial resolution. Only a comprehensive understanding of neuromodulator dynamics, their release probability, and spatial distribution will unravel their ultimate role in cognition and behavior. This Perspective offers an overview of potential design strategies for class C GPCR-based biosensors. It briefly highlights current applications of GPCR-based biosensors, with a primary focus on class C GPCRs and their unique structural characteristics compared with other GPCR subfamilies. The discussion offers insights into plausible future design approaches for biosensor development targeting members of this specific GPCR subfamily. It is important to note that, at this stage, we are contemplating possibilities rather than presenting a concrete guide, as the pipeline is still under development.

In general, techniques for monitoring neurotransmitter activity can be divided into nongenetically encoded methods (including electrophysiological methods, microdialysis, and electrochemical methods) and genetically encoded biosensors.^1^ Considering several limitations of nongenetically encoded methods, such as high invasiveness (e.g., microdialysis), low temporal resolution, and molecular specificity (e.g., fast-scan cyclic voltammetry (FSCV)), genetically encoded biosensors are an alternative to measure neurotransmitter dynamics in vivo in the behaving animal. Due to the fact that the majority of receptors for neurotransmitters and neuromodulators are G-protein-coupled receptors (GPCRs) with conserved structural topology and high specificity for endogenous neurotransmitters, GPCR-based biosensors are promising candidates to generate a library of sensors that detect most chemical signals.^2−5^

## GPCR Structure and Classes

**Figure 1:**
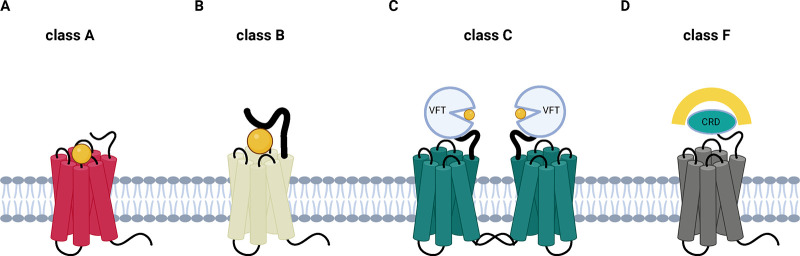
Different classes of GPCRs. Schematic of different structures of GPCRs. (A) In class A the ligand binds to the 7TM (shown in orange). (B) in class B it binds to the 7TM and the N-terminal extracellular domain. (C) In class C the ligand is bound by the Venus fly trap (VFT) domain. (D) in class F the ligand is bound by the cysteine rich domain (CRD) of the Frizzled receptor.

The superfamily of GPCRs can be divided into four classes: class A (rhodopsin-like), class B (secretin and adhesion), class C (glutamate), and class F (Frizzled)^6,7^ (Figure 1). All GPCRs share a common structure characterized by an extracellular N-terminus and seven transmembrane domains that span the membrane and are connected by three extracellular and three intracellular loops. The assembly concludes with an intracellular C-terminus.^8−10^

Class A, also named “rhodopsin-like family”, is the largest and most diverse GPCR subfamily.^11,12^ This subfamily consists in humans of 719 members that can be divided into several subgroups, such as aminergic, peptide, protein, lipid, melatonin, nucleotide, sensory, and orphan receptors.^13^ Considering the structural features of this subfamily, the first distinguishing feature is the presence of the DRY motif located at the boundary between transmembrane domain 3 (TM3) and intracellular loop (IL) 2, together with the NSxxNPxxY motif in TM7.^14^ The DRY motif is highly conserved in all rhodopsin-like receptors. This feature is valuable in the design of GPCR-based sensors by helping identify the optimal insertion site for the fluorescent protein. Class B GPCRs of the secretin subfamily comprise only 15 receptors that bind peptide hormones,^15^ whereas members of the adhesin subfamily contain 33 members.^13^ Compared to class A, the majority of receptors in this family contain conserved cysteine residues that form a cluster of cysteine bridges in the N-terminus^14^ and the N-terminal domain is relatively long in comparison to class A receptors.^13^ The presence of these cysteine residues has the potential to influence the interaction with the ligand, thereby affecting the affinity of the receptor. This, in turn, may lead to changes in the activation-induced conformational mechanism of class B GPCR-based biosensors. Regarding ligand binding, in the case of class B GPCRs, the ligand is recognized by both the extracellular and 7 TM domains. In contrast, for class A GPCRs, the endogenous ligand is recognized by a ligand binding site in the 7 TM region^16^ (Figure 1A,B). Despite the aforementioned structural differences between the two classes, the outward movement of TM6 upon ligand binding remains consistent across class A and class B, allowing the use of the same design approach for GPCR-based biosensors as described in Patriarchi et al. and Sun et al.^2,3,5^

In recent years, several biosensors based on GPCR class A and class B have been developed.^17−19^ Examples include dopamine,^2,3,20−23^ fluid sheer stress,^24^ norepinephrine and epinephrine,^25,26^ somatostatin,^27^ cholecystokinin,^27^ corticotropin releasing factor,^27^ neuropeptides,^28,27^ neurotensin,^27^ nociceptin/orphanin-FQ,^29^ vasoactive intestinal peptide^27^ ghrelin,^27^ GLP-1,^30^ urocortin,^27^ parathyroid-hormone-related-peptide,^27^ orexin and hypocretin^27^ orexin-A and orexin-B,^28^ oxytoci,^31,32^ substance P^27^ serotonin,^33−36^ and histamine.^37^ An overview of existing biosensors can be found for example in Kubitschke et al. (2024) and Rohner et al. (2024).^17,38^

Class C GPCRs bind amino acids, ions, and sugar molecules such as glutamate and γ-aminobutyric acid (GABA)^6^ and play important roles in many physiological processes, including synaptic transmission, taste sensation, and calcium homeostasis.^39^ The human subfamily contains eight glutamate receptors, two heterodimeric GABA_B_ receptors, one calcium sensing receptor, three taste receptors, a l-α-amino acid receptor, and five orphan receptors^6,13^

A unique feature of the class C subfamily is a large N-terminal domain. This domain is also responsible for constitutive homo- or heterodimerization of these receptors.^40^ Except for lycine receptors, the N-terminal extracellular domain consists of a Venus flytrap that forms the orthosteric binding site (Figure 1C) and a cysteine-rich domain that mediates signaling between the extracellular and transmembrane domains.^39,40^ The unique dimer-based composition of class C GPCRs could be a major challenge in the design of class C GPCR-based biosensors. Another critical aspect is a very low sequence similarity and the lack of conserved motifs to other GPCR classes.^39,41^ This poses a challenge to the interpretation of results from multiple sequence alignments, making it difficult to identify conserved residues. As a result, the first step in the design approach for the development of GPCR-based biosensors, specifically the determination of the insertion site for the fluorescent protein, will become challenging.^2,3,42^ It becomes evident that the complexity of the structure and the resulting low similarity of class C GPCRs with other GPCR classes necessitate the development of new design strategies for GPCR-class C-based biosensors. Particularly noteworthy is the need for attention in developing biosensors for the inhibitory members of class C GPCRs. This is crucial as these receptors play a significant role in the pathophysiology of various psychological and neurodegenerative disorders, such as GABA_B_ and GPR158 in major depressive disorder (MDD).^43−45^

Class F refers to atypical GPCRs that are mainly important for Wnt signaling in the adult and during embryonic development.^46,13^ All of these frizzled receptors share a conserved cysteine rich domain (Figure 1D), which is capable of binding to Wnt glycoproteins.

## GPCR-Based Biosensor Design Workflows

The design of a single wavelength genetically encoded biosensor is performed in several steps (for detailed review, see refs (17) and (38)). We briefly illustrate the principle of design and optimization. The general design principle of GPCR biosensors is based on conformational changes between TM5 and TM6 that occur upon ligand binding.^47^ First, a suitable GPCR scaffold is selected as the sensing moiety and for further optimization. One important aspect is the insertion site of the fluorescent protein, which plays a crucial role in the construction of a functional biosensor and can determine its functionality or failure. It is important to note that the connection site of the sensing part to the fluorescent protein (FP) is consistently found in a specific region of the protein structure.^48^ This region includes two gate posts and a flexible bulge, which allows for the insertion and fusion of sensing domains without disrupting the protein’s function.^48^ The gate posts and bulge correspond to specific residues in the GFP domain and are conserved across FP homologues.^48^ Different fluorescent proteins including circularly permuted forms (cp) such as cpGFP, cpYFP, or cpmApple can be used to build sensors that cover a wider spectral range.^4^ When selecting the insertion site, it is crucial to avoid affecting amino acid residues involved in the binding of the analyte.^49^ One way to determine the insertion site is through sequence alignments with sequences from the same class of GPCRs, as it was done in the design of the dopamine biosensor dLight, where sequences of DRD1 and DRD4 were aligned with the β2 adrenergic receptor.^2^ Today, possible insertion sites can also be identified by sequence alignments with already established GPCR-based sensors.^17,35^ Next, IL3 of the respective receptor or parts of IL3 are replaced by a cpFP.^2,3^ An advantage of this approach is the potential decoupling of the GPCR from its native intracellular binding partners, resulting in the abolition of intracellular signaling within the cell, as has been for example demonstrated in the development of GRAB and dLight sensors for dopamine.^2,3^ Typically, the inserted fluorescent protein is flanked by linker sequences at the N- or C-terminus of the fluorescent protein. These linker sequences can be optimized to improve function and expression or to increase the dynamic range in response to the ligand. The first approach for optimization is to vary the length of the linkers. In general, linkers should be kept as short as possible to maximize coupling between the GPCR and the FP. However, they should not be so short that they interfere with the folding of the protein.^50^ If the linkers are longer, there is a greater chance that the conformational change will be weakened or lost. This can happen due to bond rotations in linker residues that are not close to the chromophore. It may be advantageous to introduce deletions or utilize homologous forms with shorter flexible regions, as this may increase the association between fluorescence changes and conformational changes induced by ligand binding.^19,49^ Another option for optimization is to vary the amino acid composition by using random mutagenesis. In this approach, libraries are screened in which residues are randomized to all possible amino acids, either individually or in pairs.^50^

Due to their high probability of being essential to the fluorescence response mechanism, it is advisible to screen gate post positions first.^48^ Following this, subsequent libraries should start with the next closest residues and progress toward the sensing domain.^48^ These chimeras will then be evaluated for membrane trafficking, dynamic range, affinity, and selectivity. Mutational screening targeted within the fluorescent protein or the GPCR can be used to further increase the brightness, expression, dynamic range, specificity, or affinity of the sensor.^28^ An example for that is the study conducted by Marvin et al., where they obtained a family of high-signal-to-noise single-wavelength genetically encoded indicators for maltose.^51^ They showed that the ligand-binding properties and sensor color can be changed independently by mutating the residues in the binding site or the chromophore, respectively.^51^

**Figure 2:**
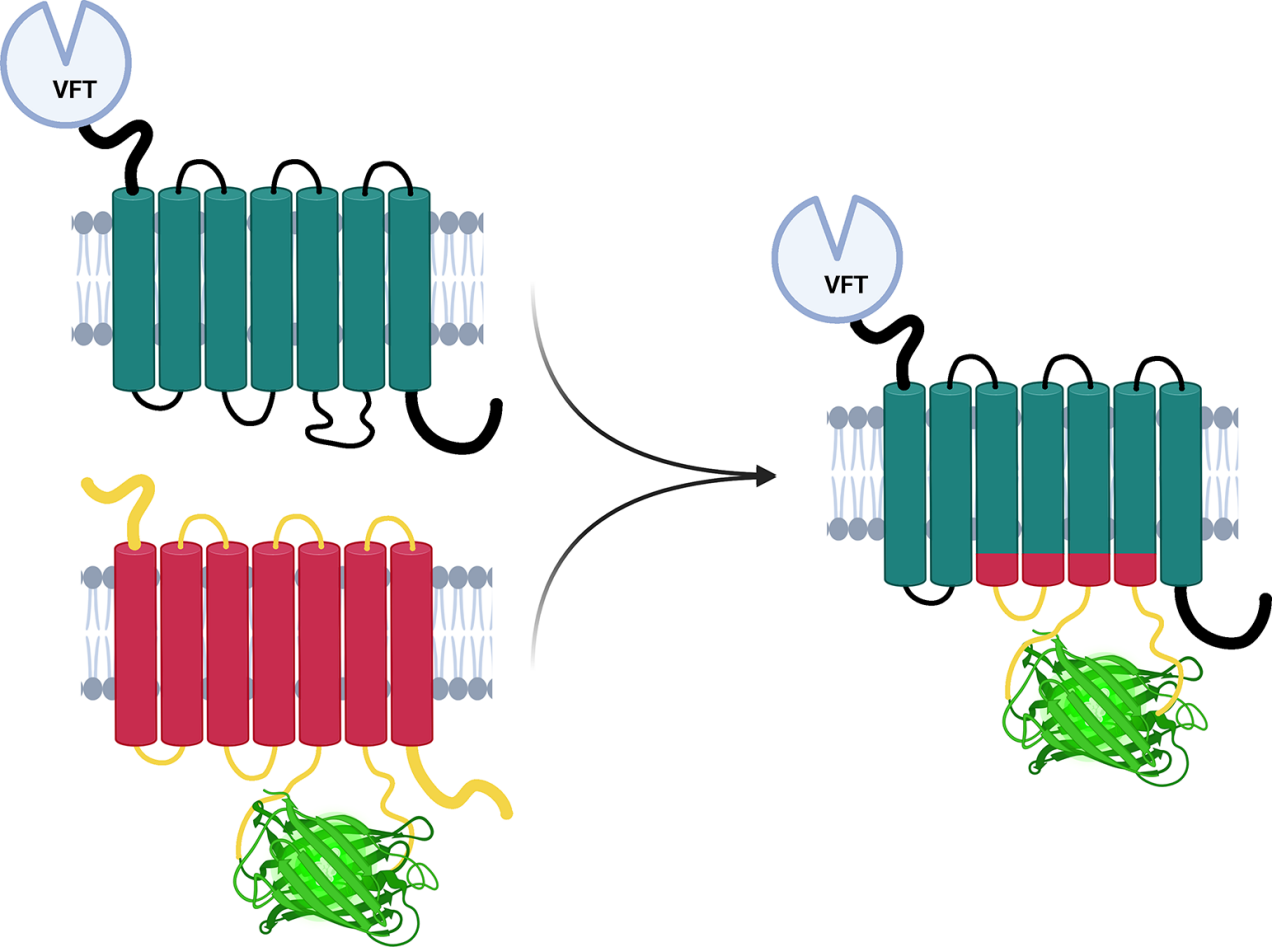
Grafting approach to design biosensors. Schematic of the design principles of the grafting approach.

In addition, GPCRs of different species can be screened to identify a good starting point for sensor generation or to improve an existing sensor. This technique was successfully used in the development of the GRAB_HA_ sensor^33^ and the new generation of multicolor norepinephrine sensors nLight.^26^ To reduce the time to develop GPCR-based biosensors, an innovative grafting approach (Figure 2) has been developed^31,33^ in which the optimized fluorescence module of an already existing GPCR based biosensor substitutes only the third intracellular loop or both the third the second intracellular loop of the native GPCR.^31,33^ By screening oxytocin receptors (OX) from several species, Ino et al. not only developed MTRIAOT, a G-protein-coupled-receptor-based green fluorescent OX sensor with a large dynamic range, appropriate affinity, and ligand specificity for OX orthologs, but they also showed the feasibility of the grafting approach for additional 46 ligands and GPCRs.^31^ Soon afterward Kagiampaki et al.^26^ used the grafting approach to substitute the second and third intracellular loops of several native GPCRS, such as muscarinic, serotonergic, and acetylcholinergic receptors, with either dLight1.3b or RdLight1 to obtain not only green fluorescent biosensors but also red-shifted sensors for multicolor imaging.^26^ Although the fluorescence change of these sensors was variable for each sensor and further improvement via mutational screening may be required, the use of this approach can drastically reduce the time required to generate new GPCR-based sensors capable of measuring specific ligands.^26^ This demonstrates the general transferability of the grafting technique to multiple receptor types.^26,31^ In the future, new optimization approaches for GPCR-based biosensors could be considered, such as structure-guided protein engineering, to increase the affinity of the ligand binding pocket in various GPCRs.

## Exploring Future Avenues in the Generation of Class C GPCR-Based Biosensors

Class C GPCRs regulate many important physiological functions. They contain receptors for the major excitatory and inhibitory neurotransmitters: glutamate and GABA. These receptors play a crucial role in the pathophysiology of various diseases. Examples include Alzheimer’s disease, amyotrophic lateral sclerosis, and Huntington’s disease. Hence, class C receptors are significant targets for drug development and the GPCR-based biosensor design. Precise visualization of neurotransmitter dynamics in health and disease would contribute to a better understanding of the molecular interplay underlying the associated diseases.^52,53^ For example, two notable ligands in this context could be γ-aminobutyric acid (GABA) and glycine, which have been shown to be involved in the pathophysiology of major depressive disorder (MDD)^45,54,55^

For GABA, a bacterial periplasmic binding protein (PBP) based fluorescent biosensor (iGABASnFR) already exists. iGABASnFR was obtained by structure-guided mutagenesis and library screening of a PBP from *Pseudomanas* fluorescence,^56^ based on the general design of the fluorescent glutamate biosensor iGluSnFR.^57^ iGABASnFR might have some advantages over a GPCR based biosensor: For example, the likelihood of iGABASnFR interacting with native GABA receptor subunits is low compared to a GPCR-based GABA biosensor. In addition, iGABASnFR shows good membrane localization and does not alter the cellular physiology.^56,57^ However, one potential problem of PBP-based biosensors is in general their limited sensitivity to detect low levels of neurotransmitter release, as indicated by a dissociation constant (*K*_d_) of approximately 9 μM for iGABASnFR.^56^ So far, also the reported off kinetics could be an obstacle to visualize fast GABA transients, as it has been reported for iGLuSnFR.^57,58^ In contrast, GPCR based biosensors could overcome limitations in binding affinities and will be highly specific for the ligand of interest. In addition, their mammalian origin will improve expression, trafficking, and possible side effects.

GABA, is the major inhibitory neurotransmitter in the brain and mediates its effects through two distinct classes of receptors: the ionotropic GABA_A_ receptors and the metabotropic GABA_B_Rs.^54^ GABA_B_ receptors are Gi-coupled receptors and are heterodimers composed of two subunits, GABA_B1_ and GABA_B2_, with very different cellular functions: The GABA_B1_ subunit is responsible for ligand binding, whereas the GABA_B2_ subunit facilitates translocation of the receptor dimer to the cell surface and activates the G-protein signaling cascade.^55^ Various techniques, including epigenetics, post-mortem studies, and analysis of GABA levels in cerebrospinal fluid and plasma, have been used to explore the role of the GABA system in the pathophysiology of MDD.^43^ Individuals suffering from depression show lower levels of GABA in both plasma and cerebrospinal fluid compared to healthy individuals.^44^

**Figure 3:**
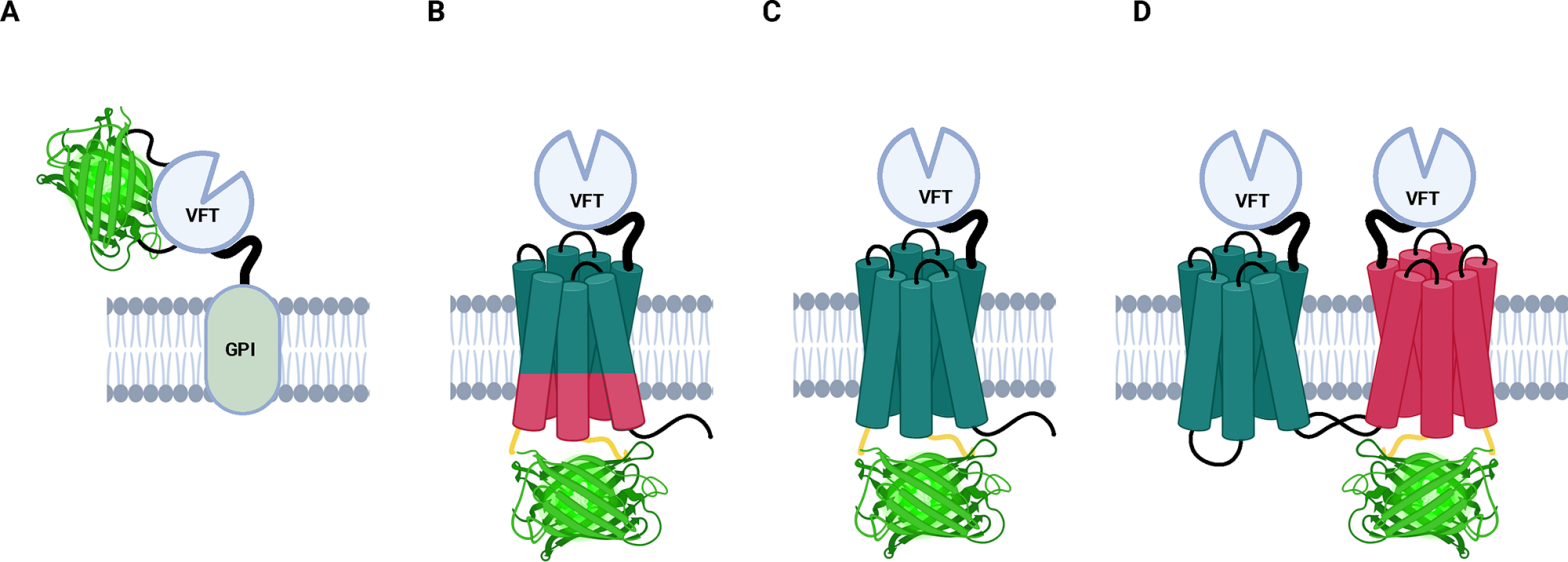
Hypothetical class C-based biosensor design. (A) GABA_B1_ N-terminal design. The fluorescent module (shown in green) is inserted into the N-terminal domain of GABA_B1_, and a GPI-anchor ensures membrane trafficking. (B) Chimera of N-terminal parts of GABA_B1_ and C-terminal parts of GABA_B2_. The fluorescence module is inserted into the ICL3 of (C) GABA_B2_. The fluorescence module is inserted into the ICL3 of GABA_B1_. (D) GABA_B2_ is used as a backbone for the insertion of the fluorescence module, which is inserted into the ICL3 of GABA_B2_, and natural dimer formation with GABA_B1_ will ensure membrane trafficking. Yellow linkers indicate linkers that can be targeted for optimization.

Given that GABA_B1_ is responsible for binding to GABA, it may be reasonable to use this subunit or parts of it as the sensing moiety in the genetically encoded biosensor. However, a potential problem that may arise in this context is the intracellular aggregation of the GABA_B1_ subunit, which may occur due to the absence of the GABA_B2_ subunit, which is crucial for membrane trafficking. A potential solution is provided in a study by Calver et al., in which they showed that removal of the entire C-terminal intracellular domain of GABA_B1_ resulted in its expression at the plasma membrane. This finding suggests that the coiled-coil region of the C-terminal domain of GABA_B1_ plays a role in retaining this subunit within the endoplasmic reticulum (ER).^60^ In addition, fusion of the GABA_B1_ subunit with glycosylphosphatidylinositol (GPI) (Figure 3A), which is a lipid anchor for many cell surface proteins could be used for membrane targeting.^61,62^ GPI-anchored proteins are a type of membrane protein consisting of a soluble protein attached to the outer leaflet of the plasma membrane by a conserved glycolipid anchor.^63^ GPI anchoring has been used in the development of an eLACCO1.1, an intensiometric green fluorescent genetically encoded biosensor for the detection of extracellular l-lactate.^64^ In this study, Nasu et al. demonstrated that the combination of a CD59-derived N-terminal leader sequence and a CD59-derived GPI anchor resulted in the desired targeting of eLACCO1.1 to the cell surface.^64^ It is important to mention that when referring to the N-terminus, only the Venus flytrap motif, not the entire receptor, is meant. Another example of GPI anchoring can be found in a study by Burgstaller et al., in which they developed a ratiometric biosensor to study local pH dynamics within subcellular microstructures in living cells. They showed that by fusing pH-Lemon to a GPI anchor, it became possible to monitor pH changes within the entire luminal space of the secretory pathway and the extracellular leaflet of the plasma membrane^65^ (Figure 3A).

Given the unique dimer-based activation mechanism of class C GPCRs, the challenge of intracellular aggregation of one of the subunits, such as GABA_B1_ in the case of GABA_B_, could be addressed by creating chimeric constructs (Figure 3B). The goal is to combine the functions of each subunit into a single genetic construct. This approach aims to produce a more compact molecular entity that contains all of the necessary functions to achieve an appropriate level of functionality. In the case of GABA_B_, the aforementioned strategy of fusing the N-terminus and heptahelical portion of GABA_B1_ with the C-terminal portion of GABA_B2_ could be considered as a next approach to improve membrane trafficking. For example, it has already been shown in the study by Pagano et al. that a truncated fragment of the C-terminus of GABA_B2_ with a deletion of the C-terminal 121 residues of the zipper domain, CR2P820, very efficiently exports GABA_B1_ to the cell surface.^66^ The intracellular retention of GABA_B1_ may be solved by fusion with a specific signal sequence, which can translocate the sensor unit to the cell membrane and ensure extracellular recognition of the ligand. In both prokaryotes and eukaryotes, signal sequences play a key role in the targeting and membrane insertion of secretory and membrane proteins. Signal sequences are usually N-terminal extensions that modulate the translocation of nascent or completed proteins from the cytosol to the plasma membrane of bacteria, the membrane of the ER in eukaryotic cells, the inner membrane of mitochondria, or the thylakoid membrane of chloroplasts.^67^ Gallegos et al. showed that by fusing appropriate targeting sequences to the NH_2_ or COOH terminus of the C-kinase activity reporter (CKAR), a genetically encoded fluorescence resonance energy transfer-based reporter for PKC activity can be targeted to the plasma membrane, Golgi, cytosol, mitochondria, or nucleus.^68^ Another example of using signal sequences as a tool to target the genetically encoded biosensors to their cellular localization was demonstrated by Palmer et al. where they targeted the genetically encoded calcium indicators to mitochondria by incorporating multiple repeats of the tandem signal sequence of human cytochrome C oxidase.^69^ An alternative approach could be to fuse the signal sequence of the GABA_B2_ subunit with the GABA_B1_ subunit. This fusion could be used to direct the genetically encoded biosensor to the cell membrane surface since one of the known functions of GABA_B2_ is to facilitate the translocation of GABA_B1_ to the plasma membrane. However, a question that may arise is the choice of which terminus to fuse the sequence in order to ensure proper targeting to the cell membrane. In some cases, it may also be necessary to consider post-translational modifications such as myristylation or palmitoylation.

Two different approaches to insert the reporting fluorescent protein could be considered: The first one is nowadays widely used^2,3^ and inserts the fluorescent protein (e.g., eGFP, cpGFP, sfcpGFP) into ICL3, whereupon ligand binding large conformational changes occur between TM5 and TM6^4,8,70^ (Figure 3C).

The second insertion site is based on the publication by Malitscheck et al. where they provided evidence suggesting that the N-terminus of the metabotropic GABA_B_ receptor is sufficient for ligand binding.^71^ A design strategy that utilizes only the N-terminus of the GABA_B_ receptor in conjunction with a membrane anchor, such as a GPI, may be promising. An advantage would be the compactness of the biosensor, which at the same time would improve its translocation from the intracellular space to the surface of the cell membrane (Figure 3A).

Another approach would use only the GABA_B2_ subunit and insert the fluorescent protein into ICL3 of GABA_B2_. This insertion site would prevent its natural functions and abolish the activation of the intracellular G-protein signaling cascade. Heterodimerization of the chimeric GABA_B2_ with the native GABA_B1_ would still facilitate translocation of GABA_B1_ to the cell surface and utilize the sensing moiety of GABA_B1_ (Figure 3D). Consequently, the resulting genetic construct could bind GABA through GABA_B1_, translating ligand-binding induced changes into conformational changes in GABA_B2_. This could presumably lead to detectable changes in the intensity of the sensor fluorescence, without intracellular aggregation of such a genetic construct.^59^ When employing GABA_B2_ and utilizing dimerization strategies, it is essential to acknowledge the potential significant impact on endogenous receptors; this possible drawback needs to be investigated in detail when obtaining a GPCR based on this design strategy.

Glycine is an inhibitory neurotransmitter but can be excitatory in developing neurons.^45^ In addition to ligand-gated glycine channels, there is an orphan receptor, GPR158, which was identified as a metabotropic receptor for glycine.^45^ Glycine appears to play a role in the pathophysiological changes observed in MDD. Plasma from patients with MDD contains decreased levels of glycine and increased levels of taurine.^72^ Exposure of mice to chronic stress increased GPR185 levels in a glucocorticoid-dependent manner^73^ and ablation of GPR185 promoted resilience, leading to the conclusion that GPR158 is a key factor in determining an individual’s vulnerability or resilience.^73^

Given that GPR158 is a homodimeric receptor,^74^ issues with different functions of the subunits that were mentioned in the previous paragraphs discussing the challenges in designing a GABA_B_-based genetically encoded biosensor for GABA do not arise.

One of the key difference between GPR158 and other class C GPCRs is that its N-terminus lacks the Venus flytrap module necessary for ligand binding and receptor activation. Instead, it possesses a cache domain, which is a known receptor for amino acids and other related small molecules that are ubiquitously used by bacterial chemoreceptors.^45,74^

The generation of chimeras, which has already been suggested as a potential strategy for the creation of GABA_B_-based biosensors mentioned earlier in the review (see Figure 3B), could be an appropriate approach; these chimeras could, for example, combine the N-terminus and the heptahelical part of GPR158 with the C-terminal part of a well-trafficked GPCR. This approach has already been used in a study by Jain et al. where they improved the trafficking of the adenosine A1 receptor (A1R) by creating chimeric receptors containing the seven transmembrane domains of the A1R and the full-length or truncated C-terminus of the A2aR.^74^ The chimeric receptors showed improved localization to the plasma membrane and were able to bind radioligand with native A1R affinity.^75^

Sequences from receptors with highly efficient membrane trafficking capabilities, such as 5-HT2C, M3R, D2R, or A2aR receptors, could be used to improve membrane targeting.^3,25,34,76^

Not only is the correct membrane targeting essential, but also proteins should not be accumulated in the ER or Golgi apparatus to prevent possible cell death or toxic reactions. Gradinaru et al. investigated this systematically^77^ and introduced a toolbox to control for trafficking of proteins with in the cell.^77^ It will be crucial to first address this issue before proceeding to the next steps in biosensor development, such as optimizing linkers and tuning the affinity.^3^

Another important consideration in the design of the GPR158-based sensor is that GPR158 possesses two additional conserved elements not typically found in class C receptors: a calcium-binding EGF-like domain (aa 314–359) and a leucine repeat region (aa 108–136).^78^ These unique features could potentially affect the determination of the insertion site for the fluorescent protein when using the Ballesteros–Weinstein algorithm.^42^ The Ballesteros–Weinstein algorithm numbering scheme can provide information about the relative position of each amino acid (AA), the AA present at that position, and the actual AA number in a given GPCR.^42^ These three numbers associated with each AA position are called identifiers.^42^ Each AA identifier starts with the transmembrane helices (TMH) number, e.g., 4 for TMH4, and is followed by the position relative to a reference residue among the most conserved AA in that TMH.^42^ This conserved AA is usually identified by the number 50.^42^ In addition to sequence alignment approaches, it is beneficial to consider other strategies to identify the insertion site such as a structure-based alignment approach. Specifically, in the case of designing structures for class C GPCR-based biosensors, a thorough investigation comparing them with known structures, such as GABA_B_ and mGluR5, using prediction tools such as Alphafold and trRosetta, is needed.^79,80^ In this context, it can be speculated that due to the rapid development of machine learning approaches in recent years, new and more precise prediction tools will soon be available on the market, which will facilitate the design of biosensors. Nevertheless, when applying multiple sequence alignments to a member of class C GPCRs, it is advisible to compare sequences within the same subfamily. This approach allows the detection of conserved regions among class C members with a higher degree of accuracy. It also facilitates the search for a suitable insertion site for the fluorescent protein. It will be important to recognize that when using the grafting approach to generate biosensors for class C GPCRs, the precautions related to multiple sequence alignment mentioned above must be considered. Careful attention to these precautions is essential to ensure the accuracy and reliability of the biosensor development process for class C GPCRs. One potential source for obtaining a reliable and comparable set of sequences is the alignment provided in the study by Jeong et al. This alignment can be used as a starting point for generating an interpretable multiple sequence alignment. By using the data from the Jeong et al. study, a foundation can be established for aligning sequences in a manner that ensures accuracy and comparability, laying the groundwork for further analysis and interpretation.^74^

## Conclusion

In this Perspective, we have proposed some potential design strategies for the development of class C GPCR-based biosensors. While the current GPCR workflow has proven successful in the development of a number of biosensors for class A and class B GPCRs, such as dopamine^2,3,20,9^ or serotonin,^33−36^ there is a recognized need to improve and adapt the current design workflow for the development of class C GPCR-based biosensors. The first precaution that should be considered prior to any design approach is the generation of an interpretable multiple sequence alignment for class C GPCRs. In general, a further understanding of the structure and function of class C GPCRs will be fundamental for the successful development and adaptation of design strategies from class A and class B GPCRs to class C GPCRs.
